# Surveillance of nasal *Staphylococcus aureus* in patients undergoing breast surgery

**DOI:** 10.1128/spectrum.03049-24

**Published:** 2025-05-23

**Authors:** Ma Jesús Pérez-Granda, Andrés Visedo, Martín Olivares, Álvaro García-Cañal, Marta Díaz-Navarro, Raquel Carrillo, Teresa Vicente, Patricia Muñoz, María Guembe, José Ma Lasso

**Affiliations:** 1 Department of Clinical Microbiology and Infectious Diseases, Hospital General Universitario Gregorio Marañón 16483, Madrid, Spain; 2 Instituto de Investigación Sanitaria Gregorio Marañón 559924 , Madrid, Spain; 3 CIBER Enfermedades Respiratorias-CIBERES (CB06/06/0058), Madrid, Spain; 4 Universidad Complutense de Madrid 16734, Madrid, Spain; 5 Department of Plastic Surgery, Hospital General Universitario Gregorio Marañón 16483, Madrid, Spain; Icahn School of Medicine at Mount Sinai, New York, New York, USA

**Keywords:** breast implant, diagnosis, molecular techniques, surveillance, nasal carriage, *Staphylococcus aureus*

## Abstract

Screening and decolonization programs have proven effective in reducing the frequency of *Staphylococcus aureus* infections, mainly in orthopedic and cardiac procedures. Despite being classified as clean, breast surgery is associated with infection. Using culture and polymerase chain reaction (PCR) assay, we aimed to assess the frequency of nasal carriage of *S. aureus* in patients undergoing breast surgery. We conducted a prospective observational 10-month study at a large tertiary teaching hospital, including patients undergoing breast reconstruction surgery who met the eligibility criteria and signed the informed consent document. Nasal swabs were collected before surgery from patients with no signs of *S. aureus* infection and tested using both culture and the Xpert MRSA/SA SSTI PCR assay. The outcomes were nasal colonization by *S. aureus*, colonization rates at the time of surgery, infection, and length of hospital stay. We included 100 patients, 27% of whom were colonized. Of these, 20 patients were positive by culture and 27 by PCR. The median (IQR) age was 56.0 (49.0–63.7) years. A total of 6 patients had infection, and the median (IQR) number of days until onset of infection was 191 (186.25–197.00). Carriage of *S. aureus* before surgery was microbiologically confirmed in two patients. We demonstrated that 27% of patients undergoing breast surgery were nasal carriers of *S. aureus*, with PCR assay being the best diagnostic strategy. Future studies are needed to address the efficacy of bacterial decolonization of the nose and nipples to reduce the frequency of infection and complications.

**IMPORTANCE:**

We showed that almost 30% of women undergoing breast surgery are nasal carriers of *S. aureus*, and PCR molecular technique was the best diagnostic tool. However, future studies are needed to implement decolonization of nasal and nipple to reduce infection and complications.

## INTRODUCTION

*Staphylococcus aureus* infections arise from the ability of the microorganism to attach to both devices and tissues, forming a biofilm that confers resistance to antibiotics and to the patient’s immune response (1, 2). The presence of additional associated virulence factors means that *S. aureus* has one of the highest associated morbidity and mortality rates (3).

Although many of the risk factors for postoperative infections are uncontrollable, nasal carriage of *S. aureus* is a risk factor that can be addressed before surgery. Staphylococci from the skin flora are commonly cultured from surgical site infections following breast reconstruction. Moreover, the presence of methicillin-resistant *S. aureus* (MRSA) has been associated with increased morbidity. *S. aureus* is usually identified using bilateral nasal swab sampling followed by culture, molecular techniques, or both (1–3).

Screening and treatment programs have been shown to decrease the incidence of MRSA and postoperative infections. Exogenous infections occur in 15% of cases and are prevented using sterile equipment, rigorous hygiene standards, and hand washing. However, carriers of *S. aureus* are more likely to develop *S. aureus* infection than non-carriers (4, 5). Consequently, eradication of carriage appears to be a rational strategy for control of *S. aureus* infection and can be achieved with antimicrobials and/or antiseptics (6). In a randomized trial examining surgical site infection, rapid detection of *S. aureus* followed by decolonization of nasal and extranasal body sites with mupirocin and chlorhexidine significantly reduced the frequency of deep infection by 80% (7). Our research has mainly been in orthopedic, spinal, and cardiac surgery (8–12).

Breast surgery is considered clean, with infection being one of the most frequent complications of postmastectomy reconstructions, occurring in approximately 2%–2.5% of cases (13). However, rates are significantly higher in postoperative infections, reaching 29% after breast reconstruction (14). Most acute and subacute infections are caused by gram-positive bacteria, with *S. aureus* being one of the most common microorganisms (15–17). The main manifestation associated with infection caused by bacterial biofilm adhesion is capsular contracture, recorded in 31.9% of cases in a recent prospective study conducted at our center (18). The paucity of evidence in the literature underscores the urgent need for an evidence-based set of “best practices” in breast surgery (19). The study by Dassoulas et al. demonstrated that the use of a standard evidence-based perioperative protocol was associated with a significant decrease in infection rates among patients undergoing implant-based breast reconstruction and was especially effective for gram-positive infections (20).

Furthermore, nipple bacterial flora has recently been shown to be associated with an increased risk of capsular contracture. Therefore, preoperative nipple bacterial flora analysis could provide useful information for clinicians treating clinically diagnosed postoperative infections (21).

The absence of data in the literature on the rate of nasal colonization by *S. aureus* in patients undergoing breast surgery led us to assess nasal carriage before and after surgery by culture and molecular techniques.

## MATERIALS AND METHODS

Ours was a prospective observational study performed in a large tertiary teaching institution in Spain. Our 1,300-bed center serves a population of 715,000 inhabitants in Madrid, Spain. The 11-bed Plastic Surgery Department, which performs approximately 500 breast surgeries per year, serves a population of 1,300,000 inhabitants.

Over a 10 month period (February to November 2023), we included all patients admitted to the Plastic Surgery Department for breast reconstruction surgery meeting the following criteria: age >18 years, signature of the informed consent form, and no clinical or microbiological signs suggestive of *S. aureus* infection. On the days of the visit for the preoperative work-up, nasal swabs were obtained according to standard clinical practice and underwent two laboratory diagnostic techniques: conventional culture and Xpert MRSA/SA SSTI polymerase chain reaction (PCR) assay (Cepheid, Spain). No patient underwent any procedure outside standard clinical practice. Samples for culture were processed on Mannitol Salt 2 agar plates (MSA2, bioMérieux, Spain). PCR samples were processed in an Xpert SA Nasal Complete cartridge (GeneXpert, Cepheid, Spain) according to the manufacturer’s instructions. The amplification cycle threshold was recorded for positive samples, as were the frequency of *S. aureus* colonization, in-hospital days, and mortality rate.

We also recorded the following clinical-demographic variables: age, sex, reason for admission, underlying disease, McCabe score, Charlson comorbidity index, and type of surgery.

### End points

- Primary: frequency of nasal colonization by *S. aureus* as determined by comparing culture and PCR as diagnostic methods
- Secondary: rate of colonization by *S. aureus* (MSSA and MRSA) at the time of surgery

### Definitions

*Nasal colonization by S. aureus (gold standard*): a positive PCR result, or a positive culture result *with S. aureus* in nasal swabs, or both. Patients were followed up until 7 months or death.

### Statistical and clinical analysis

Continuous variables are expressed as mean (SD) or median (IQR); categorical variables are expressed as continuous variables and as percentages. Categorical variables were evaluated using the chi-square test or a two-tailed Fisher exact test. Statistical significance was set at *P* <0.05 (two-tailed).

The statistical analysis was performed using IBM SPSS Statistics for Windows, Version 21.0 (IBM Corp, Armonk, New York, USA).

We calculated the validity values of culture and PCR of nasal samples by comparing patients with infection of any etiology and patients with *S. aureus* infection. The sensitivity, specificity, positive predictive value, and negative predictive value were expressed with their 95% CI and calculated using EPIDAT 3.1.

## RESULTS

A total of 233 breast surgeries were performed during the study period (10% bilateral).

**Fig 1:**
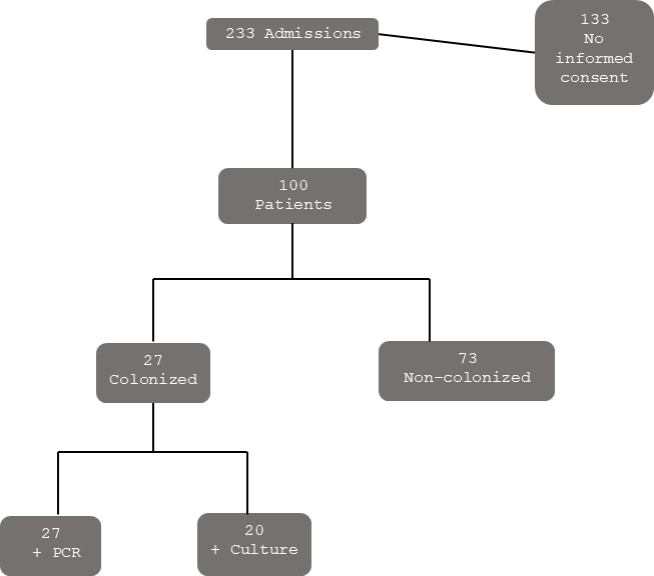
Study algorithm. PCR, polymerase chain reaction.

**Fig 2:**
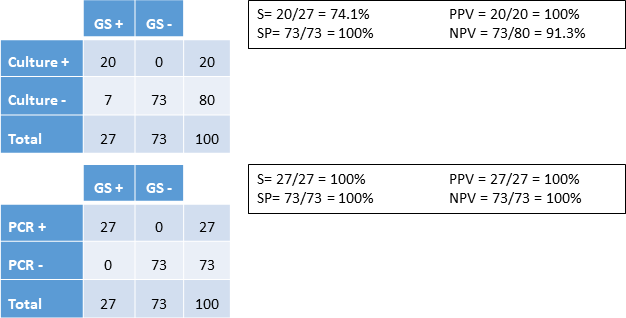
Validity values of each diagnostic technique for prediction of colonization by *S. aureus.* S, sensitivity; SP, specificity; PPV, positive predictive value; NPV, negative predictive value; GS, gold standard; PCR, polymerase chain reaction. Gold standard = positivity with any of the techniques.

Of the 100 patients, 27 (27%) were colonized according to our definition (positive culture, positive PCR, or both in the nasal sample at the time of surgery) (Fig. 1). Of these, 20 patients were positive by culture (1 MSSA and 19 MRSA) and 27 by PCR, indicating that PCR had better sensitivity and negative predictive values than conventional culture (100% vs 74.1% and 100% vs 91.3%) (Fig. 2).

Table 1 describes the characteristics of the study population (100 patients). The median (IQR) age was 56.0 (49.0–63.7) years. The main underlying conditions were peptic ulcer disease (8.0%), diabetes mellitus (5.0%), and renal dysfunction (3.0%).

**TABLE 1:** Characteristics of the study population^*a*^

Characteristic	Total *n* = 100	Colonized *n* = 27	Non-colonized *n* = 73	*P*
Median (IQR) age in years	56.0 (49.0–63.7)	52.0 (44.0–65.0)	59.0 (49.5–63.5)	0.11
Underlying conditions, N (%)				
Peptic ulcer disease	8 (8.0)	1 (3.7)	7 (9.6)	0.67
Diabetes mellitus	5 (5.0)	2 (7.4)	3 (4.1)	0.61
Renal dysfunction	3 (3.0)	2 (7.4)	1 (1.4)	0.17
Central nervous system disease	1 (1.0)	0 (0.0)	1 (1.4)	1.00
Chronic obstructive pulmonary disease	1 (1.0)	0 (0.0)	1 (1.4)	1.00
Median (IQR) hospital stay, days	3.0 (2.0–4.0)	3.0 (2.0–5.0)	2.0 (2.0–3.5)	0.18

^*a*^ IQR, interquartile range.

A total of 6 patients had infection of any etiology, and the median (IQR) number of days until onset of infection was 191 (186.25–197.00). Two patients were shown to carry *S. aureus* before surgery based on microbiological confirmation by culture.

Table 2 shows the validity values of culture and PCR for predicting infection of any etiology and *S. aureus* infection. Sensitivity was 50.0% and 100%, respectively.

**TABLE 2:** Validity values of each technique for predicting infection^*a*^

	S% (95% CI)	SP% (95% CI)	PPV% (95% CI)	NPV% (95% CI)	Validity index (95% CI)	Prevalence (95% CI)	LR+ (95% CI)	LR− (95% CI)
Infection of any etiology								
Nasal PCR	50.00 (1.66–98.34)	74.47 (65.12–83.81)	11.11 (0.00–24.82)	95.89 (90.65–100.00)	73.00 (63.80–82.20)	6.00 (0.85–11.15)	1.96 (0.82–4.68)	0.67 (0.30–1.51)
Nasal culture	50.00 (1.66–98.34)	81.91 (73.60–90.23)	15.00 (0.00–33.15)	96.25 (91.46–100.00)	80.00 (71.66–88.34)	6.00 (0.85–11.15)	2.76 (1.11–6.86)	0.61 (0.27–1.37)
*S. aureus* infection								
Nasal PCR	100.00 (75.00–100.00)	74.49 (65.35–83.63)	7.41 (0.00–19.14)	100.00 (99.32–100.00)	75.00 (66.01–83.99)	2.00 (0.00–5.24)	3.92 (2.79–5.50)	–
Nasal culture	100.00 (75.00–100.00)	81.63 (73.46–89.81)	10.00 (0.00–25.65)	100.00 (99.38–100.00)	82.00 (73.97–90.03)	2.00 (0.00–5.24)	5.44 (3.59–8.26)	–

^*a*^ S, sensitivity; SP, specificity; PPV, positive predictive value; NPV, negative predictive value; LR, likelihood ratio; CI, confidence interval; PCR, polymerase chain reaction.

## DISCUSSION

We found that *S. aureus* had colonized the nose in 27% of patients undergoing breast surgery and that PCR was superior to culture for predicting nasal colonization by *S. aureus*. Moreover, 7.4% of *S. aureus* nasal carriers eventually developed *S. aureus* infection.

*S. aureus* infection, particularly in patients undergoing surgery, poses a significant risk owing to the microorganisms’ ability to form biofilms, thus contributing to antibiotic resistance and immune evasion. Nasal carriers of *S. aureus* are at a higher risk for postoperative infections, especially MRSA, which is associated with increased morbidity (1, 2).

Screening and decolonization programs using mupirocin and chlorhexidine have proven effective in reducing infections, although primarily in orthopedic and cardiac procedures (7–12). Breast surgery, despite being classified as clean, is associated with high infection rates, particularly after reconstruction, with *S. aureus* frequently implicated (13–17). The use of a standard evidence-based perioperative protocol was recently shown to significantly decrease infection rates among patients undergoing implant-based breast reconstruction and was especially effective for gram-positive infections (20).

We found that PCR was more sensitive than conventional culture for prediction of colonization (100% vs 74.1%). In the study by Muñoz et al., 28.5% of the 200 patients who were to undergo major heart surgery were colonized by *S. aureus*. Both culture and PCR results were positive in 33 patients, and only PCR results were positive in 24 patients (9). When we analyzed the sensitivity and negative predictive value of each diagnostic technique for predicting infection by any microorganism or by *S. aureus*, both techniques yielded similar values (50.0% and 100%, respectively). However, results were slightly worse for PCR in terms of specificity and positive predictive value than culture because swab cultures could only be obtained in two out of the six patients with infection. Therefore, we are unable to determine whether *S. aureus* was the cause of infection in any of the remaining four episodes. In addition, PCR could have shown better validity values for prediction of *S. aureus* infection.

Screening of various body sites to detect *S. aureus* remains controversial. While Troeman et al. found *S. aureus* colonization at multiple body sites to be an independent risk factor for *S. aureus* infection (4, 5), Bouza et al. (31 patients colonized by *S. aureus* admitted for major heart surgery) found that lower respiratory tract samples were only positive in 3 patients, with the remaining 28 being either positive in the nasal sample or positive in both samples (22). Therefore, it seems that screening for nasal carriage of *S. aureus* (and, if possible, other microorganisms) could be useful for decolonization programs. In patients undergoing breast surgery, it may also be important to assess colonization of the nipple, in addition to the nose, by *S. aureus*.

Another important aspect is that, at present, despite the remarkable rate of *S. aureus* carriage (1–3), reported rates of decolonization before surgery are quite low. In the study by Muñoz et al., only 21% of colonized patients had undergone an attempt to decolonize before the surgical intervention, consistent with the findings of Troeman et al. (24.7%) (5, 9). Consequently, it is necessary to introduce evidence-based protocols for screening and decolonization of *S. aureus* carriers—at least in nasal carriers—among candidates for surgery.

Despite our study providing new information regarding *S. aureus* carriage in breast surgery, it has some limitations. It was a 1 year observational study with a small sample size and conducted at a single center, so a larger cohort may be needed to validate our results and enhance the generalizability of the findings to a broader population. Its observational design captures a snapshot of colonization rates at a single time point, so future longitudinal studies could provide more comprehensive insights into the dynamics of *S. aureus* colonization and infection. Moreover, despite considering that our results are reliable enough to demonstrate that *S. aureus* infection was mainly caused by previous *S. aureus* colonization, we did not assess other variables that may also contribute to infection development.

### Conclusion

We demonstrated that 27% of patients undergoing breast surgery were nasal carriers of *S. aureus* and that PCR was the best diagnostic strategy. Future studies are needed to address the efficacy of decolonization of nasal and nipple bacteria to reduce infection and complications and to establish evidence-based protocols for screening and decolonization.

## AFTER EPUB

*[This article was published on 23 May 2025 with an error in the Acknowledgments. The Acknowledgments were corrected in the current version, posted on 30 May 2025.]*
